# Influence of Non-Thermal Atmospheric Pressure Plasma Treatment on Shear Bond Strength between Y-TZP and Self-Adhesive Resin Cement

**DOI:** 10.3390/ma12203321

**Published:** 2019-10-12

**Authors:** Dae-Sung Kim, Jong-Ju Ahn, Eun-Bin Bae, Gyoo-Cheon Kim, Chang-Mo Jeong, Jung-Bo Huh, So-Hyoun Lee

**Affiliations:** 1 Department of Prosthodontics, Dental Research Institute, Dental and Life Science Institute, BK21 PLUS Project, School of Dentistry, Pusan National University, Yangsan 50612, Korea; modesthanks@gmail.com (D.-S.K.); tarov0414@hanmail.net (J.-J.A.); 0228dmqls@hanmail.net (E.-B.B.); cmjeong@pusan.ac.kr (C.-M.J.); neoplasia96@hanmail.net (J.-B.H.); 2 Department of Oral Anatomy and Cell Biology, School of Dentistry, Pusan National University, Yangsan 50612, Korea; ki91000m@pusan.ac.kr; 3 Research & Development Center, FEAGLE Corporation, Yangsan 50614, Korea

**Keywords:** yttria-stabilized tetragonal zirconia polycrystal (Y-TZP), plasma treatment, shear bond strength, self-adhesive resin cement

## Abstract

The purpose of this study was to evaluate the effect of non-thermal atmospheric pressure plasma (NTP) on shear bond strength (SBS) between yttria-stabilized tetragonal zirconia polycrystal (Y-TZP) and self-adhesive resin cement. For this study, surface energy (SE) was calculated with cube-shaped Y-TZP specimens, and SBS was measured on disc-shaped Y-TZP specimens bonded with G-CEM LinkAce or RelyX U200 resin cylinder. The Y-TZP specimens were classified into four groups according to the surface treatment as follows: Control (no surface treatment), NTP, Sb (Sandblasting), and Sb + NTP. The results showed that the SE was significantly higher in the NTP group than in the Control group (*p* < 0.05). For the SBS test, in non-thermocycling, the NTP group of both self-adhesive resin cements showed significantly higher SBS than the Control group (*p* < 0.05). However, regardless of the cement type in thermocycling, there was no significant increase in the SBS between the Control and NTP groups. Comparing the two cements, regardless of thermocycling, the NTP group of G-CEM LinkAce showed significantly higher SBS than that of RelyX U200 (*p* < 0.05). Our study suggests that NTP increases the SE. Furthermore, NTP increases the initial SBS, which is higher when using G-CEM LinkAce than when using RelyX U200.

## 1. Introduction

Yttria-stabilized tetragonal zirconia polycrystal (Y-TZP) is one of the most widely used restorative materials along with Computer-Aided Design and Computer-Aided Manufacturing (CAD-CAM) development in the dental field [1]. Y-TZP is known as a biocompatible ceramic with outstanding aesthetics, low thermal conductivity, and chemical stability [2,3]. In particular, Y-TZP has superior compressive and flexural strength as compared with glass and feldspar ceramics [4].

In the case of conventional ceramics, micromechanical retention with hydrofluoric acid and chemical bonding through silane coupling agents increase bonding strength with resin cement [5,6]. However, these methods are usually considered not efficient for Y-TZP because of its high corrosion resistance due to the polycrystalline structure containing little silica [7,8,9]. In order to overcome this drawback, a combination of surface roughening by sandblasting and chemical adhesion using self-adhesive resin cement has been introduced [8,10]. Sandblasting is a common method of providing micromechanical interlocking between Y-TZP and resin cement [11,12,13,14]. Self-adhesive resin cement, unlike conventional resin cement that requires an additional adhesive system, is useful for bonding to zirconia prosthesis with simple application and low postoperative sensitivity [3,15,16]. Moreover, phosphate monomers contained in self-adhesive resin cements help to form chemical bonds with Y-TZP surfaces. [3,13,16]. Previous studies have reported that the use of sandblasting and functional monomer-containing resin cement shows reliable clinical bonding to zirconia ceramic [9,12,13,14,17]. However, until now, there has been no definitive protocol for the most effective bonding to zirconia prosthesis [10,18,19].

Plasma-surface modification has been used in many industrial applications for decades [20]. Plasma is considered the fourth state of matter and occurs naturally in the sun, stars, lightning, and aurora [2,21]. Plasma includes large amounts of highly reactive species such as ions, electrons, free radicals, and electronically excited neutrons [4,21]. These activated plasma species produce a reactive surface without significantly changing the bulk properties of the materials [22,23]. The plasma treatment has also been applied to the dental field and can be a suitable method for increasing zirconia adhesion [24,25]. Previous studies have compared the effects of sandblasting and non-thermal atmospheric pressure plasma (NTP) on shear bond strength (SBS) between Y-TZP and resin cement [26,27]. However, all of these studies used only conventional resin cements and did not confirm any thermocycling effect.

The purpose of this in vitro study is to evaluate the effect of NTP on SBS between Y-TZP and self-adhesive resin cement in different surface treatments. We performed surface energy (SE) analysis using contact angle after Y-TZP surface treatment. In our SBS test, two types of self-adhesive resin cements (G-CEM LinkAce (GC Corporation, Tokyo, Japan), RelyX U200 (3M ESPE, St. Paul, MN, USA)) were used, and the thermocycling effect was assessed. Furthermore, we observed failure mode by optical microscope and field emission scanning electron microscopy (FE-SEM) after the SBS test.

## 2. Materials and Methods 

### 2.1. Preparation of Y-TZP Specimens

Twelve cube-shaped Y-TZP specimens (10 × 10 × 10 mm^3^) were prepared for surface energy analysis and 160 disc-shaped Y-TZP specimens (5 mm in diameter, 3 mm in thickness) were prepared for the SBS test. The manufacturing process was done by milling pre-sintered Y-TZP blocks (LUXEN, DentalMax, Seoul, Korea) and sintering according to the manufacturer’s instructions. The disc-shaped Y-TZP specimens for the SBS test were embedded in acrylic resin (Orthodontic Resin, DENTSPLY Caulk, Milford, DE, USA) and the surfaces were exposed for cement bonding. All the Y-TZP specimens were polished with 600 grit and 800 grit silicon carbide paper at 300 rpm for 60 s using a grinding machine (MetaServ 250, Buehler, Lake Bluff, IL, USA).

The surface treatments applied to the Y-TZP specimens are as follows: No surface treatment (Control), non-thermal atmospheric pressure plasma treatment (NTP), sandblasting (Sb), sandblasting followed by non-thermal atmospheric pressure plasma treatment (Sb + NTP). 

**Figure 1:**
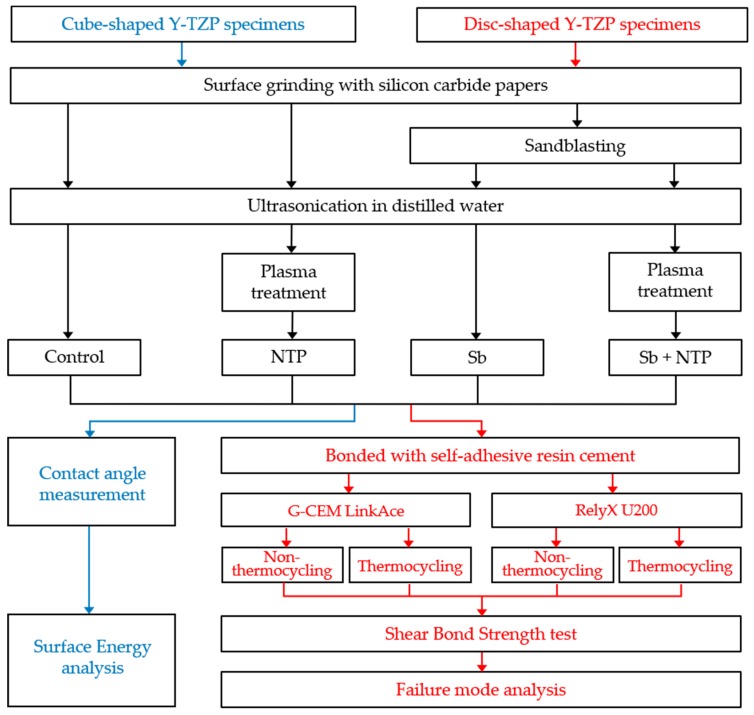
Flowchart of the experimental process.

The sandblasting treatment was carried out vertically for 15 s, with a pressure of 2.5 bar using a dental sandblaster (Basic master, Renfert, Hilzingen, Germany). This process was performed at 10 mm away from the Y-TZP surface using particles of 50 μm aluminum oxide (Al_2_O_3_) (Hi-Aluminas, Shofu Inc., Kyoto, Japan). After sandblasting, all the Y-TZP specimens were ultrasonically cleaned in distilled water for 3 min and then dried with an air syringe. Figure 1 summarizes the overall procedure, and Table 1 shows the number of specimens distributed across the entire experimental group.

### 2.2. Non-thermal Atmospheric Pressure Plasma (NTP) Treatment

**Figure 2:**
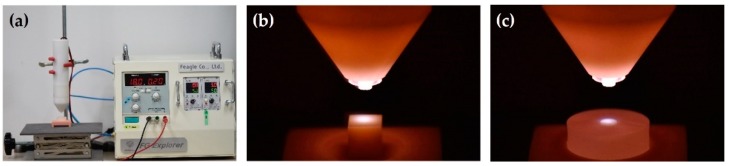
(**a**) Non-thermal atmospheric pressure plasma (NTP) device; (**b**) Plasma treatment on cube-shaped Y-TZP specimen; (**c**) Plasma treatment on disc-shaped Y-TZP specimen embedded in acrylic resin.

For the NTP treatment, a plasma generating device (FG-Explorer, FEAGLE, Yangsan, Korea) was used. The working gas was argon with the flow rate of 5.0 slm (standard liter per minute), the peak-to-peak applied voltage was 3 kV_pp_, and the peak plasma discharge current was 4.80 mA. NTP generated in this machine does not extend the plasma plume outwardly like a plasma jet, but instead it forms the plume inside the plasma source. The tip of the plasma source was positioned at 10 mm above the surface of the Y-TZP specimen and placed vertically so that the NTP could be applied to the entire surface, and the treatment was performed for 1 minute (Figure 2).

### 2.3. Contact Angle Measurement and Surface Energy (SE) Analysis

All of the cube-shaped Y-TZP specimens were distributed three per surface treatment group. Two different test liquids were used for the contact angle measurement: the polar liquid water, and the dispersive liquid diiodomethane. The contact angle was measured using a sessile drop technique with a computer-controlled image analyzer equipped with a video camera (Ramé-Hart 190-U1, Ramé-Hart Instrument Co., Succasunna, NJ, USA). After dropping each solvent once per specimen, the contact angles of the liquid droplets were calculated by averaging values of the left and right sides. The SE of each group was calculated by the Owens-Wendt method from the measured contact angle values. In this method, the SE of Y-TZP was calculated as the sum of the two components given in the Equation (1):

(1) $$\gamma_{s}={\gamma_{s}}^{p}+{\gamma_{s}}^{d}$$

where, $\gamma_{s}$—the SE of Y-TZP,

${\gamma_{S}}^{p}$—the polar component of the SE of Y-TZP,

${\gamma_{S}}^{d}$—the dispersive component of the SE of Y-TZP.

The polar component of the SE of Y-TZP was obtained from the Equation (2):

(2) $${\gamma_{s}}^{p}={\left(\frac{\gamma_{w}(\cos\theta_{w}+1)-2\sqrt{{\gamma_{s}}^{d}{\gamma_{w}}^{d}}}{2\sqrt{{\gamma_{w}}^{p}}}\right)}^{2}$$

and the dispersive component of the SE of Y-TZP was obtained from the Equation (3):

(3) $${\gamma_{s}}^{d}={\left(\frac{\gamma_{d}(\cos\theta_{d}+1)-\sqrt{\frac{{\gamma_{d}}^{p}}{{\gamma_{w}}^{p}}}\gamma_{w}(\cos\theta_{w}+1)}{2\left(\sqrt{{\gamma_{d}}^{d}}-\sqrt{{\gamma_{d}}^{p}\frac{{\gamma_{w}}^{d}}{{\gamma_{w}}^{p}}}\right)}\right)}^{2}$$

where, $\gamma_{w}$—the SE of water,

${\gamma_{w}}^{p}$—the polar component of the SE of water, 

${\gamma_{w}}^{d}$—the dispersive component of the SE of water,

$\gamma_{d}$—the SE of diiodomethane, 

${\gamma_{d}}^{p}$—the polar component of the SE of diiodomethane,

${\gamma_{d}}^{d}$—the dispersive component of the SE of diiodomethane,

$\theta_{w}$—the contact angle of water,

$\theta_{d}$—the contact angle of diiodomethane.

### 2.4. Shear Bond Strength (SBS) Test

#### 2.4.1. Fabrication of Y-TZP Specimens Bonded with Self-Adhesive Resin Cement Cylinders

**Figure 3:**
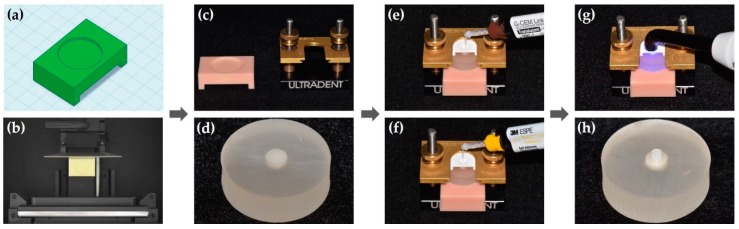
A series of the bonding process. (**a**) CAD data and (**b**) 3D printing for the positioning stand; (**c**) 3D printed positioning stand and bonding clamp; (**d**) Disc-shaped Y-TZP specimen embedded in acrylic resin; (**e**) Apply G-CEM LinkAce and (**f**) RelyX U200 to the Y-TZP surface using a plastic jig; (**g**) Light curing with an LED curing light; (**h**) Disc-shaped Y-TZP specimen bonded with the resin cylinder.

**Figure 4:**
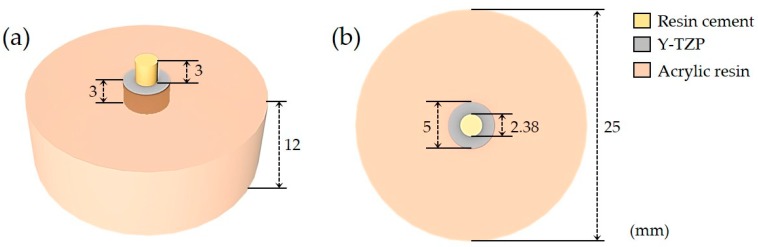
Three-dimensional schematic image of the Y-TZP specimen for the Shear Bond Strength (SBS) test. (**a**) Top-front view and (**b**) top view of the specimen.

Two self-adhesive resin cements, G-CEM LinkAce (GC Corporation, Tokyo, Japan) and RelyX U200 (3M ESPE, St. Paul, MN, USA), were used for the SBS test. The information on these cements is shown in Table 2. All of the disc-shaped Y-TZP specimens were distributed 40 per surface treatment group. For each group, half of the specimens were bonded with G-CEM LinkAce resin cylinder, and the rest were bonded with RelyX U200 resin cylinder. Each resin cylinder was made in a uniform size by injecting self-adhesive resin cement into a ready-made plastic jig (Ultradent Jig, Ultradent Products Inc., South Jordan, UT, USA) with a diameter of 2.38 mm and a height of 3 mm, then light polymerized at 1000–1200 mW/cm^2^ for 20 seconds in three directions with an LED curing light (Elipar™ DeepCure-L, 3M ESPE, St. Paul, MN, USA). At this time, a custom-made positioning stand was used for the Y-TZP specimens embedded in acrylic resin. This stand was manufactured with a CAD program (Tinkercad, Autodesk Inc., San Francisco, CA, USA) and a 3D printer (DIO PROBO, DIO inc., Busan, Korea) (Figure 3 and Figure 4). 

#### 2.4.2. Thermocycling Process and Shear Bond Strength (SBS) Measurement

After polymerization, half of the disc-shaped Y-TZP specimens bonded with resin cement cylinder were stored in distilled water at 37 °C for 24 h before the SBS test. The rest were aged by thermocycling. Thermocycling was carried out for 5000 cycles at 5–55 °C, the interval between the baths was 2 s, and the residence time was 30 s per temperature. Subsequently, SBS was measured at a 1.0 mm/min crosshead speed using a testing machine (Shear Bond Tester, Bisco Inc., Schaumburg, IL, USA) at the maximum load until failure. This SBS measurement method followed the International Organization for Standardization (ISO 29022:2013) [28].

### 2.5. Failure Mode Analysis

The surfaces of SBS tested Y-TZP specimens were observed at a magnification of 40 × using an optical microscope (BX51, Olympus, Tokyo, Japan). Failure modes were classified as an adhesive failure occurring between Y-TZP and the resin cement, cohesive failure occurring in resin cement, and mixed failure where adhesive and cohesive failures occur at the same time. Representative debonded specimens were plated with platinum (Quorum Q150T S, Quorum Technologies Ltd., Ashford, Kent, UK) and evaluated with FE-SEM (Supra 25, Zeiss, Jena, Germany).

### 2.6. Statistical Analysis

One-way analysis of variance (ANOVA) was used to analyze the effects of surface treatment on the SE and the SBS, and post-hoc tests were performed with Dunnett T3. The SBS values in accordance with the cement type and the thermal effect were compared using the independent sample t-test (*p* < 0.05). All the statistical analyses were performed using a software program (SPSS Statistics V24, IBM Corp., Chicago, IL, USA).

## 3. Results

### 3.1. Contact Angle Measurement and Surface Energy (SE) Analysis

**Figure 5:**
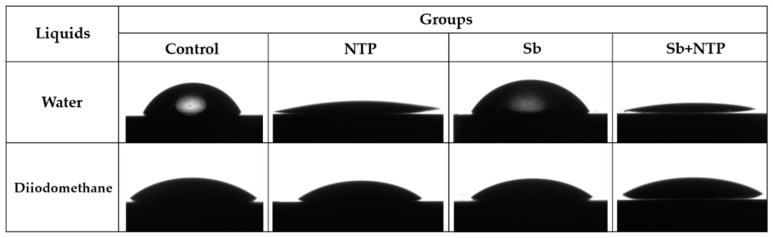
Images of contact angles of water and diiodomethane droplets on different Y-TZP surface treatments. Control, no treatment; NTP, non-thermal atmospheric pressure plasma; Sb, sandblasting; Sb + NTP, sandblasting followed by non-thermal atmospheric pressure plasma.

**Figure 6:**
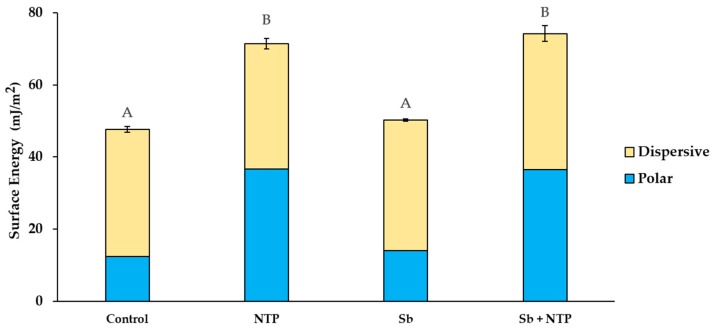
Means of polar and dispersive components of SE of Y-TZP for the Control, NTP, Sb, and Sb + NTP group. The sum of the two components represents the value of SE of Y-TZP. The different upper case letters indicate significant differences among different surface treatments (*p* < 0.05).

Figure 5 is a representative image of the contact angle measured using water and diiodomethane for SE calculation. Figure 6 shows the average values of the polar and dispersive parts of SE, and the sum of these represents the value of SE of Y-TZP. The SE value of the NTP group was significantly higher than that of the Control group (*p* < 0.05). Moreover, the SE value of the Sb + NTP group was significantly higher than that of the Sb group (*p* < 0.05). In contrast, there were no significant differences between the Control and Sb groups. These results were mainly affected by the polar component rather than by the dispersive component SE of Y-TZP.

### 3.2. Shear Bond Strength (SBS) Test

**Figure 7:**
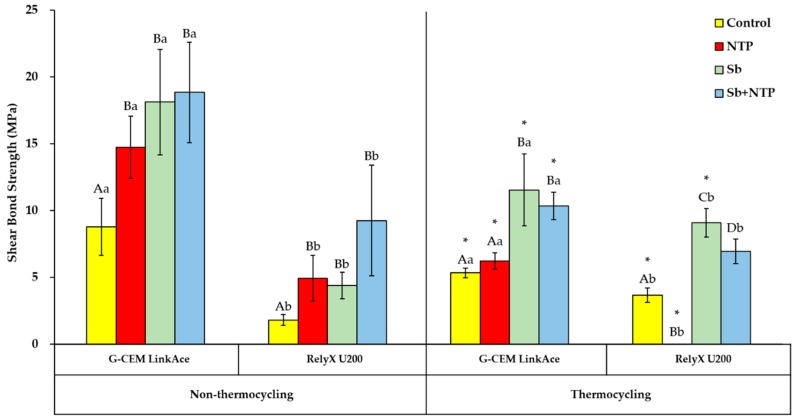
Mean SBS results of the Control, NTP, Sb, and Sb + NTP groups in accordance with the thermal effect and the type of self-adhesive resin cement. The different upper case letters indicate significant differences between surface treatments within the same thermal effect and the cement type (*p* < 0.05). The different lower case letters indicate significant differences between cement types within the same surface treatment in each thermal effect (*p* < 0.05). * indicates significant differences between thermal effects within the same surface treatment in each cement (*p* < 0.05).

Figure 7 shows the mean SBS values, standard deviations, and statistical significance of all the experimental groups. First, in accordance with different surface treatments, the SBS values were compared within the same thermal effect and cement type. For G-CEM LinkAce and RelyX U200 in non-thermocycling, respectively, the SBS value of the NTP group was significantly higher than that of the Control group (*p* < 0.05). However, there were no significant differences between the NTP, Sb, and Sb + NTP groups. For G-CEM LinkAce in thermocycling, the SBS values of the Sb and Sb + NTP groups were significantly higher than that of the Control group (*p* < 0.05). In contrast, there were no significant differences between the Control and NTP groups. For RelyX U200 in thermocycling, the SBS value of the NTP group was significantly lower than that of the Control group (*p* < 0.05). Conversely, the respective SBS values of the Sb and Sb + NTP groups were significantly higher than the SBS value of the Control group (*p* < 0.05). Moreover, in thermocycling, the respective SBS values of the NTP and Sb + NTP groups were significantly lower than the SBS value of the Sb group (*p* < 0.05). 

Second, in accordance with the thermal effect, the SBS values were compared within the same surface treatment in each cement. For G-CEM LinkAce, the respective SBS values of the NTP and Sb + NTP groups were significantly lower in thermocycling than in non-thermocycling (*p* < 0.05). For RelyX U200, the SBS value of the NTP group was significantly lower in thermocycling than in non-thermocycling (*p* < 0.05).

Third, in accordance with the type of cement, the SBS values were compared within the same surface treatment in each thermal effect. Regardless of thermocycling, the respective SBS values of the NTP and Sb + NTP groups were significantly higher in G-CEM LinkAce than RelyX U200 (*p* < 0.05). 

### 3.3. Failure Mode Analysis

**Figure 8:**
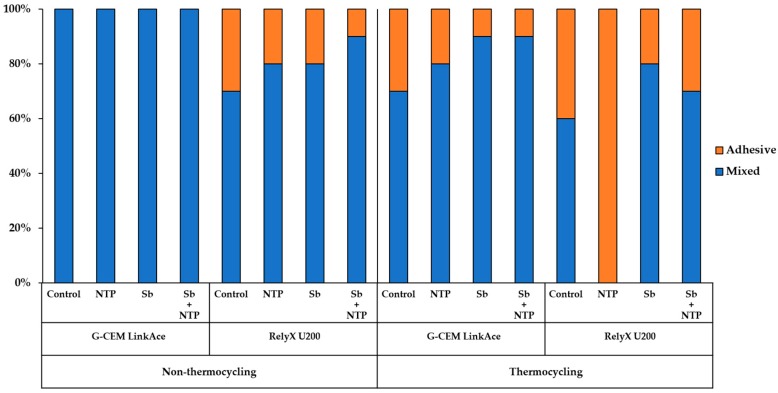
Failure mode distribution of the Y-TZP for each group after the SBS test.

**Figure 9:**
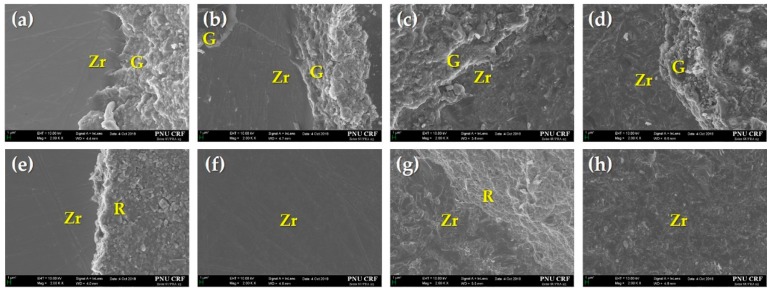
Representative FE-SEM images (2000×) of the Y-TZP surface in each group with thermocycling after the SBS test. (**a**–**d**) represent the Control, NTP, Sb, and Sb + NTP groups of G-CEM LinkAce, respectively; (**e**–**h**) represent the Control, NTP, Sb, and Sb + NTP groups of RelyX U200, respectively. (**a**–**e**,**g**) show mixed failures; (**f**,**h**) show adhesive failures. Zr, Y-TZP surface; G, fractured surface of G-CEM LinkAce; R, fractured surface of RelyX U200.

The failure modes of all the experimental groups are shown in Figure 8, and the failure pattern is recorded as a percentage. Compared with non-thermocycling, the adhesive failure rate for the Control, NTP, and Sb + NTP groups was increased regardless of the cement type in thermocycling. Regardless of thermal effects, the adhesive failure rate for each group was higher in RelyX U200 than in G-CEM LinkAce. Cohesive failure in resin cement did not occur in any group. Figure 9 shows typical FE-SEM images of SBS tested Y-TZP specimens after thermocycling. 

## 4. Discussion

Zirconia material is widely used in dentistry because of its excellent mechanical characteristics and high opacity, in particular, when blackish abutments are involved [7]. However, due to the structural nature of Y-TZP, it is difficult to obtain proper adhesion to zirconia prosthesis in dental treatment [27]. Ahn et al. [8] and Abi-Rached et al. [29] have reported that sandblasting is used to increase surface roughness, remove contaminants, and activate zirconia surfaces. Some articles also have mentioned that this mechanical surface treatment can improve the bond strength between zirconia and various types of cement [29,30]. In contrast, several studies have shown that the microporosity created by sandblasting can act as a cracking agent to worsen the long-term stability of zirconia-based restorations [4,7,22,25]. Additionally, He et al. [31] have also been mentioned that the remaining Al_2_O_3_ particles after sandblasting could degrade the adhesion, requiring the cleaning of the surface. Therefore, other surface treatment methods without physical modification would be useful for adhesion to zirconia.

Non-thermal atmospheric pressure plasma (NTP) processing is considered a promising alternative to mechanical surface treatment to increase the bond strength between Y-TZP and resin cement [32]. In contrast to sandblasting, previous studies have reported that NTP treatment has little effect on surface roughness [7,33,34,35]. This is because NTP achieves surface treatment at an electron level using an ionized inert gas [24,27]. The argon gas used in our study is the most commonly used inert gas in plasma-surface modification [20,22,26]. This gas has the advantages of low cost, low ionization energy, and high surface cleaning effect [20,22,26]. In addition, because NTP treatment does not require Al_2_O_3_ particles unlike sandblasting, specimen cleansing was not necessary after NTP processing in our experiments. Therefore, in dental clinics, we suggest that NTP treatment can be a simple method that rarely causes mechanical surface change to zirconia restorations.

Ito et al. [34] and Valverde et al. [36] have reported that NTP treatment increases the surface energy (SE) of Y-TZP. During NTP treatment, the moisture from the gas and atmosphere is decomposed by high energy electrons to generate OH radicals [24,26,27]. It breaks or removes the C-C and C-H bonds of organic impurities attached to the zirconia surface [24,26,27]. Consequently, this effect allows the formation of active peroxide radicals that augment the combination of functional groups such as C-O- and C-OH to the Y-TZP surface [4]. The increase in the polar part containing oxygen on the surface improves the surface wettability [2,21]. This is consistent with the decrease of contact angle and the increase of SE in accordance with NTP treatment in the current study.

Several studies have also reported that NTP promotes the generation of intermolecular secondary forces, such as van der Waals bonds, occurring between the hydroxyl groups present on the Y-TZP surface and in the resin cement [4,36]. These reports support the result of our study that the SBS value of the NTP group was significantly higher than that of the Control group, regardless of the cement type in non-thermocycling. However, for G-CEM LinkAce in thermocycling, there were no significant differences between the Control and NTP groups. In particular, for RelyX U200 in thermocycling, all the Y-TZP specimens of the NTP group showed early failures. This early failure means that the resin cylinder was debonded from the Y-TZP surface during thermocycling. The SBS of Y-TZP specimens with early failure was assumed to be zero value [37]. Irrespective of the cement type, the SBS values of the NTP group were significantly lower in thermocycling than non-thermocycling. These results are consistent with previous reports of a significant decrease in SBS due to thermocycling after NTP treatment [23,25,38]. Therefore, the SBS after NTP treatment may be affected by thermal stress, the compositions of resin cement, and low resistance to hydrolysis of the van der Waals bond [39,40]. Despite these findings, we suggest that NTP treatment probably helps to increase the initial SBS between Y-TZP and self-adhesive resin cement. Increased initial SBS can prevent premature debonding of zirconia prosthesis. 

NTP treatment has been reported to increase the SBS value between Y-TZP and resin cement to a level similar to sandblasting [26,27]. In the present study, for G-CEM LinkAce and RelyX U200 in non-thermocycling respectively, there were no significant differences in the SBS between the NTP and Sb groups. In addition, we confirmed the effect of the combination of two surface treatments: sandblasting followed by NTP treatment. Regardless of the cement type in non-thermocycling, the respective SBS values of the NTP and Sb groups were not significantly different from the Sb + NTP group. In other words, the effect of the combination of two surface treatments was similar to that of sandblasting or NTP treatment. By contrast, regardless of the cement type in thermocycling, the SBS value of the NTP group was significantly lower than that of the Sb and Sb + NTP groups. Besides, the SBS value of the Sb + NTP group was not significantly higher than that of the Sb group. In other words, the NTP treatment and the combination of two surface treatments were found to be not as effective as sandblasting.

In our study, we also compared the SBS between G-CEM LinkAce and RelyX U200 in different surface treatments. The manufacturer reports that G-CEM LinkAce is self-adhesive resin cement and contains distinctive ester phosphate monomers [15,41]. RelyX U200 is also a self-adhesive resin cement, including methacrylate monomers and phosphate groups [15,41]. When these monomers contact with Y-TZP, the oxygen groups of the zirconia and the hydrogen groups of the monomers gradually create water molecules and make firm covalent bonds [41]. At this time, the length of the –(CH_2_)_n_ chain of the monomer affects the bonding efficiency of the phosphoric monomers [41]. In the present study, regardless of thermocycling, all the groups of G-CEM LinkAce showed significantly higher SBS than those of RelyX U200. Therefore, although each manufacturer did not disclose the exact information, we suggest that the difference in the SBS may be due to the monomer characteristics of the cement. Moreover, the difference in viscosity between these two cements may affect their interaction with NTP treatment. G-CEM LinkAce has fewer filler particles and lower viscosity than RelyX U200 [42]. Accordingly, in our study, it is possible that G-CEM LinkAce spread more widely on the Y-TZP surface than RelyX U200 and resulted in higher SBS than RelyX U200. For this reason, we infer that the G-CEM LinkAce reacts more to the functional groups on the NTP-treated Y-TZP surface than RelyX U200.

The custom-made positioning stand used in the SBS test allowed the Y-TZP surface to be in contact with Ultradent Jig at a constant position. In the design of the study protocol, we tried to apply each cement to the Y-TZP surface using the jig without the positioning stand. However, Y-TZP specimens were not in a particular position, and some cement was found outside of the Y-TZP surface. To overcome these drawbacks, we designed and printed the stand using CAD and 3D printer to fit the size of the bonding clamp. The stand enabled the cement to be applied to the center of the Y-TZP surface so that no variables were in the SBS test.

As a result of the failure mode observation, the adhesive failure rate for each group was generally higher in RelyX U200 than G-CEM LinkAce and in thermocycling than non-thermocycling. This suggests that the SBS values are associated with the failure mode. For RelyX U200 in non-thermocycling, the mixed failure rate for the NTP group was higher than that of the Control group. However, for RelyX U200 in thermocycling, 100% of adhesive failures were observed in the NTP group. This finding is related to the premature failure during thermocycling. Moreover, it implies that the SBS value of the NTP group for RelyX U200 was the weakest among the experimental groups. 

The main limitation of this in vitro study is that surface treatment with zirconia primer was not performed. Future research should add various NTP conditions, resin cements, and adhesive systems. In addition, no surface roughness measurement was done in this study. Further investigation is needed to determine the correlation between surface roughness and SBS for different surface treatments. Also, thermocycling in our laboratory study did not provide enough water saturation to evaluate hydrolytic durability. Long-term water storage is necessary to distinguish clinically durable zirconia bonding systems from non-durable zirconia bonding systems [9,14]. In order to ensure the effectiveness of NTP treatment, it would be beneficial to measure the bond strength by fabricating specimens in the form of zirconia prosthesis used in dental clinics.

## 5. Conclusions

This study suggests that NTP treatment increases the SE of Y-TZP. According to the SBS test, regardless of the cement type in non-thermocycling, NTP treatment significantly increased the SBS between Y-TZP and self-adhesive resin cement. In terms of SBS increase, this NTP treatment effect was similar to sandblasting and the combination of two surface treatments: sandblasting followed by NTP treatment. However, regardless of the cement type in thermocycling, NTP treatment did not significantly increase the SBS. This NTP treatment showed significantly lower SBS than sandblasting and the combination of two surface treatments. Furthermore, within the same surface treatment and cement type, NTP treatment showed significantly lower SBS in thermocycling than in non-thermocycling. Within the same thermal effect and surface treatment, NTP treatment showed significantly higher SBS in G-CEM LinkAce than in RelyX U200. In conclusion, NTP treatment significantly increases the initial SBS, which is higher when using G-CEM LinkAce than when using RelyX U200.

## Figures and Tables

**Table 1:** Distribution of yttria-stabilized tetragonal zirconia polycrystal (Y-TZP) specimens for the entire experimental group. Control, no treatment; NTP, non-thermal atmospheric pressure plasma; Sb, sandblasting; Sb + NTP, sandblasting followed by non-thermal atmospheric pressure plasma.

Groups	Distribution of Y-TZP specimens (n)				
	Surface Energy (SE) analysis	Shear Bond Strength (SBS) test			
		G-CEM LinkAce		RelyX U200	
		Non-thermocycling	Thermocycling	Non-thermocycling	Thermocycling
Control	3	10	10	10	10
NTP	3	10	10	10	10
Sb	3	10	10	10	10
Sb + NTP	3	10	10	10	10

**Table 2:** Characteristics of two types of self-adhesive resin cement used in the SBS test.

Material	Manufacture	Type	Composition
G-CEM LinkAce	GC Corporation, Tokyo, Japan	Self-adhesive Dual-cure Automix	Paste A: Fluoroalumino silicate glass, Urethane dimethacrylate (UDMA), Dimethacrylate, Pigment, Silicon dioxide, Initiator, Inhibitor
			Paste B: Urethane dimethacrylate (UDMA), Dimethacrylate, Phosphoric acid ester monomer, Initiator, stabilizer
RelyX U200	3M ESPE, St. Paul, MN, USA	Self-adhesive Dual-cure Automix	Base paste: Methacrylate monomers containing phosphoric acid groups, Methacrylate monomers, Silanated fillers, Initiator components, Stabilizers, Rheological additives
			Catalyst paste: Methacrylate monomers, Alkaline(basic) fillers, Silanated fillers, Initiator components, Stabilizers, Pigments, Rheological additives
